# Involvement of Molecular Mechanisms between T/B Cells and IL-23: From Palmoplantar Pustulosis to Autoimmune Diseases

**DOI:** 10.3390/ijms23158261

**Published:** 2022-07-27

**Authors:** Takemichi Fukasawa, Asako Yoshizaki-Ogawa, Atsushi Enomoto, Kiyoshi Miyagawa, Shinichi Sato, Ayumi Yoshizaki

**Affiliations:** 1 Department of Dermatology, The University of Tokyo Graduate School of Medicine, Tokyo 113-8655, Japan; fukasawat-der@h.u-tokyo.ac.jp (T.F.); yoshizakia-der@h.u-tokyo.ac.jp (A.Y.-O.); satos-der@h.u-tokyo.ac.jp (S.S.); 2 Laboratory of Molecular Radiology, Center for Disease Biology and Integrative Medicine, The University of Tokyo Graduate School of Medicine, Tokyo 113-8655, Japan; aenomoto@m.u-tokyo.ac.jp (A.E.); miyag-tky@umin.ac.jp (K.M.)

**Keywords:** palmoplantar pustulosis, B cells, T cells, IL-23, anti-IL-23 antibody

## Abstract

Palmoplantar pustulosis (PPP) is a disease that causes recurrent blisters and aseptic pustules on the palms and soles. It has been suggested that both innate and acquired immunity are involved. In particular, based on the tonsils and basic experiments, it has been assumed that T and B cells are involved in its pathogenesis. In addition, the results of clinical trials have suggested that IL-23 is closely related to the pathogenesis. This review describes PPP and the genetic background, the factors involved in the onset and exacerbation of disease and its relation to the molecular mechanism. In addition, we describe the usefulness of biological therapy and its implications in relation to the importance in pathology, the pathogenesis of PPP, the importance of the role of the IL-23–Th17 axis and IL-36 in PPP. Furthermore, we describe an animal experimental model of PPP, the efficacy and mechanism of action of guselkumab, an anti-IL-23 antibody, the latest research, and finally the possibility for it to be effective for other autoimmune diseases.

## 1. Introduction

Palmoplantar pustulosis (PPP) is a disease that causes recurrent blisters and aseptic pustules on the palms and soles. Pustules are caused by the accumulation of neutrophils (a type of white blood cell), which are involved in the inflammatory response, beneath the stratum corneum, the top layer of the skin. The rash begins as small blisters (bullae) that gradually turn into pustules. Later, scabs (crusts) form and the stratum corneum (thin layer on the top surface of the skin) peels off. Later, the rash becomes a mixture of these lesions. At the beginning, the rash often itches. There are no bacteria (germs) or fungi (molds) in the ooze of this skin disease. Therefore, there is no infection from the hands and feet to other parts of the body. Thus far, the cause of PPP is unknown, but we will summarize the basic findings known to date. In this review, in particular, we will focus on the involvement of T/B cells and IL-23.

## 2. PPP and Molecular Genetic Background

### 2.1. Familial Onset Is Rare, but Genetic Risk Factors Are Presumed

There are few reports of familial onset of PPP [1,2]. Yanai et al. performed human leukocyte antigen (HLA) typing within a family with PPP and reported that 4 out of 6 family members with PPP had haplotypes A2, B46, Cw1, DR8, and DQ1 [1]. Matsuoka et al. reported two families with PPP and psoriasis vulgaris (PsV) in the family [2]. In one family, the grandmother had PPP, and the mother and daughter had PPP and PsV, and both had A2, B35, Cw3 DRw8, and DRw52 haplotypes. In another family, where a mother had PPP and PsV and a son had PPP, both had haplotypes of A26, Bw60, DRw9, and DRw53. PPP has also been reported from Japan with IL36RN (Interleukin 36 Receptor Antagonist) gene mutation [3] and CARD14 (Caspase recruitment domein-containing protein 14) gene mutation [4]. CARD14 mutations are also considered a risk factor in Europe [5]. However, there might be other genetic risk factors that have not yet been identified.

### 2.2. Involvement of Cytokines

Mutations in IL19, IL20, and IL24 have been reported as risk factors for PPP [6,7,8]. Missense mutations in the CARD14 gene have been reported in PPP. Recently, mutations in AP1S3 (Adaptor Related Protein Complex 1 Subunit Sigma 3) were reported. ATG16L1 (Autophagy Related 16-like 1) mutation was also detected in PPP [9]. ATG16L1 is an essential gene for autophagy. These mutations are involved in the production of antimicrobial peptides, IL-18, and IL-1. Loss-of-function mutations in IL-36RN activate the IL-36 signaling pathway, resulting in excessive cytokine production [9], which is also seen in PPP, albeit at a lower frequency [10].

In addition to environmental factors and genetic susceptibility, both innate and acquired immunity are involved in the pathogenesis of PPP. Inflammation of sweat glands within the epidermis forms blisters and pustules [11]. IL-17 expression is increased in eccrine sweat glands, suggesting that this site is important in the pathogenesis [12]. It has been suggested that IL-8 (neutrophil chemoattractant), IL-1α, IL-1β, IL-17A, IL-17C, IL-17D, IL-17F, IL-22, IL-23A, and IL-23 receptor are elevated in the lesions of PPP [13,14,15]. Increased serum levels of TNF-α, IL-17, IL-22, and IFN-γ have been detected in patients with PPP [13,14,15]. Elevated expression of IL-17, IL-8, and IL-36γ has been reported in sweat glands. Tonsil infection triggers PPP [16].

## 3. Factors involved in the Onset and Exacerbation of Disease and Its Relation to the Molecular Mechanism

Smoking, focal infections such as asymptomatic tonsillitis and asymptomatic periodontitis, severe constipation and irritable bowel syndrome, metal allergies, as well as stress are involved in the development of PPP. In treating PPP, it is necessary to be properly informed about its relationship to smoking cessation, to focal infection, and to metal allergy. In this section, the relationship of PPP to smoking, focal infection, and metal allergy will be discussed in detail.

### 3.1. PPP and Smoking

Smoking rates in PPP patients are very high, at about 80%. Smoking cessation tends to reduce symptoms [17] and improve treatment efficacy [18,19]. Possible mechanisms include direct stimulation of the tonsils by smoking which increases the production of inflammatory substances (such as interleukin (IL)-17), induces or aggravates inflammation in the tonsils, aggravates other factors such as periodontitis, alters immune responses to commensal bacteria, and activates cholinergic signaling pathways through the production of Tumor necrosis factor (TNF)-α from epidermal cells and nicotinic acetylcholine receptors.

### 3.2. PPP and Focal Infection (e.g., Lesional Tonsils, Dental Lesions)

**Figure 1:**
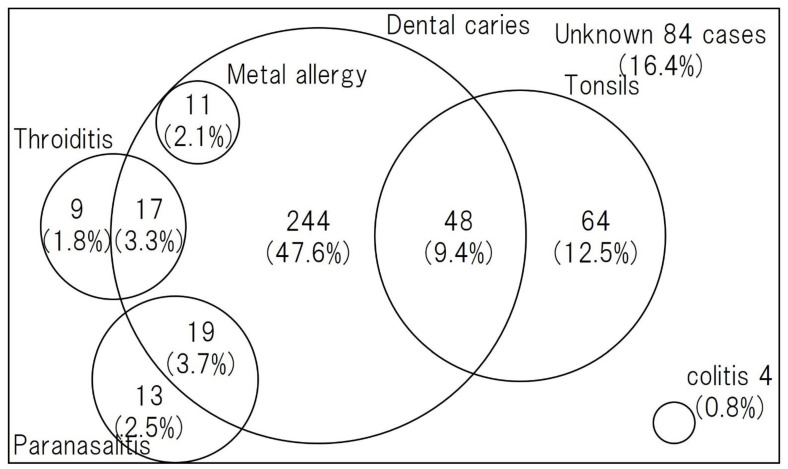
Comorbidity, including focal infection in PPP [25]. Dental caries was the most common focal infection, followed by tonsillitis and sinusitis.

Asymptomatic chronic inflammation somewhere in the body may trigger secondary inflammation in remote organs (skin, joints, kidneys, etc.) such as PPP or glomerulonephritis. This is called “focal infection”. PPP is a typical disease that is closely associated with focal infection, and conditions that usually do not require treatment, such as asymptomatic tonsillitis (focal tonsils) [20], asymptomatic periodontitis (dental foci), chronic sinusitis, nasopharyngitis, otitis media, and cholecystitis, are thought to be involved in the development and persistence of those susceptible to PPP. Due to the high efficacy of tonsillectomy, PPP, sternoclavicular hyperplasia, and IgA nephropathy are recognized as representative diseases of tonsil foci infection. Other skin diseases such as psoriasis vulgaris and allergic purpura, osteoarticular diseases such as some chronic rheumatoid arthritis and nonspecific joint pain, SAPHO syndrome (A syndrome of unexplained bone and joint symptoms and cutaneous manifestations. “SAPHO” is an acronym for Synovitis, Acne, Pustulosis, Hyperostosis, and Osteitis.), RFAPA syndrome (Abbreviation for periodic fever, aphthous stomatitis, pharyngitis, and cervical adenitis. An “autoinflammatory disease” with four main symptoms: periodic fever, aphthous stomatitis, pharyngitis, and cervical lymphadenitis, which develops during infancy.), persistent low-grade fever, and Behcet’s disease have also been reported to be associated with focal infection. More than 3/4 of cases have been reported to have focal infections or other triggers for disease onset [21] (Figure 1).

Treating these foci of infection (tonsillitis, periodontitis, sinusitis, nasopharyngitis, etc.) will lead to treatment of PPP. All of them are often asymptomatic and are not usually treated, but since they may be associated with the onset or worsening of PPP symptoms, they should be searched for in all cases and treated aggressively. If there is a possible involvement of pharyngitis/tonsillitis, the tonsils may be removed, and if periodontitis is found in or around the root of the teeth, dental treatment may improve the symptoms. If during treatment of a lesion, a temporary worsening of a skin rash or osteoarthritis is observed, it is assumed that the lesion is involved in the pathogenesis (id reaction). Transient worsening of the rash or a short interval between the onset of the disease and surgery often results in early postoperative resolution of the rash. The efficacy rate of a tonsillectomy is as high as 60–90% for skin rash in PPP and sternoclavicular arthritis in PAO (pustulotic arthro-osteitis) [22]. Pre- and postoperative smoking cessation is recommended because the smoking group shows less improvement after a tonsillectomy than the nonsmoking group. It has been speculated that nasopharyngitis may be involved in some cases in which a tonsillectomy is ineffective. A recent retrospective study showed that skin rash severity (Palmoplantar Pustulosis Area and Severity Index; PPPASI) improved significantly at 1 month after a tonsillectomy, with a high rate of improvement at 1 year postoperatively [22]. The prevalence of dental infections in PPP patients was 87–90% and 62.8–64.5%, respectively, and the treatment of dental infections was effective in about two-thirds of cases [23,24].

### 3.3. PPP and Metal Allergies

Although an association with metal allergies to dental and other metals has been reported, the percentage is as low as 5% [25], and many cases do not improve with the mere removal of dental metals [26]. Even when dental treatment is performed first and dental metal removal is performed in invalid cases, the efficacy rate is not very high at 33%, suggesting that priority should be given to dental infections first [24].

## 4. Pathogenesis of PPP

Although PPP is known as a focal infection, its pathogenesis is the activation of autoimmunity in lymphoid tissues such as the tonsils, and several mechanisms have been postulated.

**Figure 2:**
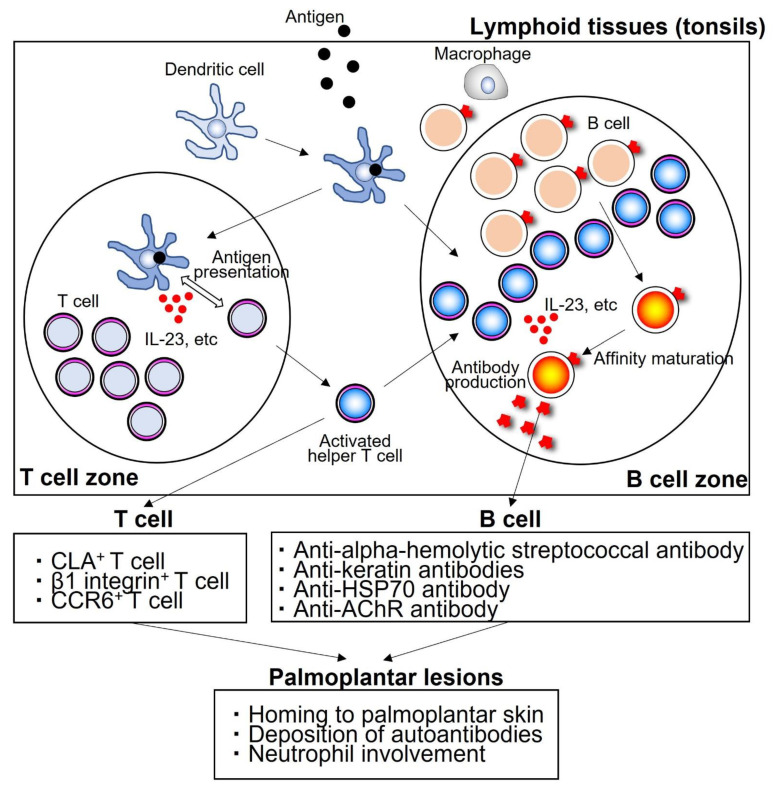
An autoimmune reaction that begins in lymphoid tissues such as the tonsils. T and B cells are activated in lymphoid tissues by cytokines such as IL-23. Activated T cells homing to the lesions on the palmoplantar surface. Activated B cells produce autoantibodies. CLA: cutaneous lymphocyte-associated antigen, CCR: C-C chemokine receptor, HSP: heat shock protein, AChR: acetylcholine receptor.

The tonsils have a crypt, but at the blind edge of the crypt, the crypt epithelium and tonsil parenchyma are mixed, and antigen-presenting cells such as dendritic cells and memory B cells are distributed, and antigen recognition is initiated. The tonsillar parenchyma is divided into lymphofollicular and interfollicular areas, the former of which activates helper T cells and B cells and promotes antibody production, and the latter of which is mainly populated by T cells and where antigen presentation from dendritic cells to naive T cells occurs. Tonsillar lymphocytes are activated upon stimulation by pathogens such as *Streptococcus pneumoniae* and *Haemophilus influenzae*, while they do not react against the commensal bacteria, alpha-hemolytic streptococci, and immune tolerance is established. However, tonsillar lymphocytes of PPP patients develop a breakdown in immune tolerance to alpha-hemolytic streptococci, resulting in an excessive immune response. The disruption of immune tolerance against commensal bacteria results in B cell activation, which induces an autoimmune response against common antigens in the tonsillar epithelium, palmoplantar skin, and alpha-hemolytic streptococci. In addition, T cells are over-activated in the lesional tonsil homing to the skin. The tonsils of PPP patients are a source of autoreactive activated T and B cells, the removal of which may lead to the suppression of the abnormal autoimmune response. Thus, in tonsillitis, it is believed that hyperimmune and autoimmune reactions are triggered [27,28,29] from a breakdown of immune tolerance to α-hemolytic streptococci (Figure 2).

In patients with PPP, TCR repertoire analysis of peripheral lymphocytes, studies of anti-keratin antibodies, and tonsils have been performed, and other mechanisms are also being considered. In the tonsils of patients, chronic inflammation caused by bacterial infection leads to a severe keratinization of the epithelium of the crypts and an increase in high molecular weight keratin, which is common to the palmoplantar epithelium, followed by the prolonged sensitization of lymphocytes of the tonsils to high molecular weight keratin and other substances in the lymphoepithelial symbionts of the crypts, resulting in the production of sensitized lymphocytes and autoantibodies. In the palmoplantar surface, CD4-positive cells, mainly TCRVβ6, infiltrate the skin and react with high molecular weight keratin and anti-keratin antibodies, activating complement and other factors, resulting in the formation of blisters and pustules (Figure 2).

In addition, genetic polymorphisms in promoter regions involved in the production of specific cytokines are thought to be involved in the pathogenesis of the disease [30]. The high blood TNF-α levels in patients with tonsil foci infection in PPP and the high cytokine levels produced by the alpha-streptococcal stimulation of excised tonsil mononuclear cells suggest that some individuals are more likely to have increased TNF-α production due to their genetic background. The disease is not necessarily caused by focal infection alone, but rather by a genetic predisposition to produce proinflammatory cytokines in response to infection, which is added to focal infection.

Similar to the classic IL-23-driven disease psoriasis, therapeutic CD20 blockade can lead to the emergence of PPP, suggesting that B cell depletion could aggravate this disease. One mechanism for this phenomenon is that CD20 antibodies eliminate most of the B cells. Therefore, as we will show later, not only bad pathogenic B cells but also good regulatory B cells are eliminated. This effect may be strongly influenced by the removal of immature B cells that regulate immune responses. Therefore, we believe that treatment that can remove only pathogenic B cells will be required in the future.

Although specific PPP genetic components have not been identified as class I antigens, class II MHC antigens do not drive PPP pathogenesis. Therefore, presumably, tissue antigens but not circulating antigen-presenting cells dominate the immune responses.

Activation of immune cells such as T cells and B cells in lymphoid tissues such as the tonsils leads to an infiltration of T cells and the deposition of antibodies in lesions such as the palmoplantar area. Neutrophils are induced and activated by cytokines and chemokines, mainly IL-17 and IL-8, produced by these T and B cells. Thus, neutrophils are activated and infiltrate into the epidermis, causing the pathogenesis of PPP in the palms and soles. Therefore, therapies targeting IL-23 and its downstream counterpart IL-17 may be effective in treating PPP. Clinical trials are actually underway that target these molecules, and they are being shown to be effective in the treatment of PPP.

## 5. Importance of the Role of IL-23–Th17 Axis and IL-36 in PPP

In recent years, the importance of IL-36 in PPP has received much attention [31]. IL-36 forms a family of three molecules, IL-36α, IL-36β, and IL-36γ. Keratinocytes in the skin rash area have been shown to express high levels of IL-36. At the same time, keratinocytes express IL-36R and produce inflammatory cytokines and chemokines via the auto-activation by IL-36, resulting in exacerbations. IL-36R is also expressed in DCs and macrophages. Moreover, its expression is increased by IL-36 stimulation.

DCs activated by IL-36 produce IL-23 and differentiate T cells into Th17 cells. IL-17A produced by Th17 cells provides stimulation to keratinocytes, and activated keratinocytes induce further IL-36 production. In fact, it has been shown that the expression levels of IL-36 and IL-17A are correlated in the skin rash area. Thus, IL-36 plays an important role in the pathogenesis, and the IL-23–Th17 axis and IL-36 are involved in the exacerbation of the skin rash by forming positive feedback.

## 6. Animal Experimental Model of PPP

No other mammals are known to spontaneously develop PPP except humans. Moreover, unlike psoriasis, there are few reports on animal models of PPP. In the following, we describe those animal models.

### 6.1. Model of PPP Tonsil Cell Transfer in SCID Mice

To directly test whether cells in the tonsils produce skin rashes, a model was created in mice with severe combined immunodeficiency (SCID). Intraperitoneal administration of mononuclear cells prepared from excised tonsils of PPP patients with worsening skin rash to SCID mice resulted in hair loss on the cheeks and forehead and the appearance of a skin rash 4–8 weeks later [32]. Human IgG was present in their serum for a long period of time, peaking after 6–8 weeks. Human anti-keratin antibodies, which are reported to be high in PPP patients, were also present, peaking at 4–6 weeks.

Epidermal thickening and blister formation were seen in the skin of the rash area, with a small infiltrate of CD4+ T cells, but few CD8+ T cells or B cells were seen. In contrast, mice transferred with peripheral blood mononuclear cells from PPP patients and mice transferred with tonsil mononuclear cells from non-PPP patients showed no skin changes. These results indicate that the tonsil cells of PPP patients can respond to the skin and produce a skin rash.

### 6.2. Model of PPP Tonsil Cell Transfer and Skin Grafting in SCID Mice

Next, an experiment was set up in which skin grafts were also used to see if the amygdala cells of PPP patients responded directly to the patient’s own palmoplantar skin. The dorsal skin of SCID mice was incised and subcutaneously grafted with rashless skin from the sole of a PPP patient, and at the same time mononuclear cells from the excised tonsils of the same patient were conditioned and administered intraperitoneally. After 2 weeks, the mouse skin covering the grafted skin was opened circularly and exposed externally, and patient skin was harvested from the mice 4 weeks later.

CD3+ T cell infiltration was seen mainly in the papillary layer of the dermis, CD4+ T cells were predominant, but there was also a large infiltration of CD8+ T cells, and human IgG deposition was seen in about half of the cases [33]. Lymphocyte function-associated antigen-1 (LFA-1) and intercellular adhesion molecule-1 (ICAM-1) were highly expressed when lymphocyte infiltration was high. In the group that received intraperitoneal peripheral blood mononuclear cells from the same patient, there was some but less T-cell infiltration, and LFA-1 and ICAM-1 expression was also less than in the tonsillar mononuclear cell transfer group of the same patient. There was little T-cell infiltration in the group that received only skin grafts and no transfer of mononuclear cells, or in the group that received skin grafts from non-PPP patients and transferred peripheral blood mononuclear cells. These results suggest that in patients with PPP, tonsillar mononuclear cells migrate to the skin of the distant palms and cause skin rashes.

### 6.3. PPP Tonsil Cell Transfer in SCID Mice and HSP Administration Model in Skin

Heat shock proteins (HSPs) are present in all cells, from bacteria to mammals, and have highly homologous amino acid sequences. They are also highly immunogenic and may be antigens of an immune response. Antibodies to bacterial HSP are elevated in PPP patients, and there have been reports of a decrease in these antibodies after the treatment of dental lesion infections and a tonsillectomy.

The above SCID mouse model also showed an increase in human anti-HSP65 IgG antibodies to bacterial HSP65 when tonsil mononuclear cells and skin grafts from PPP patients were transferred to these mice. Strong expression of human HSP60 was also observed in the skin around actual PPP lesions. Therefore, when recombinant HSP60 was administered subcutaneously on the back of SCID mice transferred with tonsil mononuclear cells from PPP patients instead of skin grafts, human anti-HSP60 IgG antibodies were raised. This indicates that PPP’s tonsillar mononuclear cells are immunoreactive to HSP60, which is expressed in autologous plantar skin. Similarly treated mice also had elevated human anti-HSP65 IgG antibodies [32]. This indicates a cross-reactivity between human HSP60 and bacterial HSP65. These results suggest that one of the mechanisms of the pathogenesis of PPP is that tonsillar lymphocytes sensitized to HSP65 from oral bacteria respond to HSP60 in the skin.

### 6.4. What We can Learn from Animal Models and the Difficulties

The above models are experimental systems that can only be performed when a tonsillectomy is performed on PPP patients, and it is quite difficult to use them, including receiving donations of rash-free skin on the plantar surface. As a control group, the excised tonsils of patients with sleep apnea and other non-PPP conditions were transferred, which could have been used because of the otolaryngology department, but would normally be difficult to obtain at the same time. In addition, although mononuclear cells from the tonsils are transferred, human neutrophils are not transferred, so the overall immune response of PPP cannot be seen, and it is considered to be a model of an early stage of the disease that begins with mononuclear cell infiltration and blistering. However, the results of this series of human tonsil cell transfer experiments in SCID mice are considered valuable data because they show how specifically involved tonsil cells are in the pathogenesis of PPP.

## 7. Efficacy and Mechanism of Action of Guselkumab, an Anti-IL-23 Antibody

In the above mechanism, IL-23, one of the inflammatory cytokines, is deeply involved in the pathogenesis of PPP (Figure 2). Since IL-23 is involved in Th17 cell differentiation and proliferation, IL-23 and Th17 cells may be deeply involved in the pathogenesis of PPP. In fact, the anti-IL-23 antibody, guselkumab, is the only biologic currently indicated for the treatment of PPP in Japan. Guselkumab 100 mg is injected subcutaneously at 0-week, 4-week, and 8-week intervals thereafter. In the randomized phase II and III studies in Japan, the primary endpoint was “change from baseline in Palmoplantar Pustulosis Area and Severity Index (PPPASI) Total Score at 16 weeks”, and the secondary endpoints were “change from baseline in PPP severity index (PPSI) at 16 weeks”, “PPPASI-50”, which is the percentage of patients with PPPASI improvement of at least 50% from baseline at 16 weeks, and “PPPASI-50”, which is the percentage of patients with PPPASI improvement of at least 50% from baseline at 16 weeks. The investigators reported significant improvements in its primary endpoint and both of the secondary endpoints [34,35] (Table 1). Data for 1.5 years have also emerged, showing sustained effects [36].

It is safe and has few side effects of Candida, which is considered to be a specific signal. There are few contraindications, and the drug is considered safe for use in the elderly.

As mentioned above, treatment of focal infection is of primary importance in the treatment of PPP and PAO, but there are cases in which this is ineffective. Early detection and early treatment are especially important for PAO, because the progression of PAO leads to progressive joint destruction. When the treatment of focal infection is ineffective, the early introduction of guselkumab may be used to prevent progression to PAO. It is closely related to its mechanism of action of suppressing Th17 cells, and its high safety profile suggests that it may be used as an anchor drug in PPP therapy. In a subanalysis of clinical trials, guselkumab was shown to be beneficial for PAO [37].

## 8. Usefulness of Biological Therapy and Its Implications of Importance in Pathology

Pharmacotherapy for PPP includes topical therapy, phototherapy, oral therapy, and biologic therapy by injection [25], and the appropriate treatment is selected according to the symptoms of the patient. The following sections describe the characteristics of each treatment method. In practice, treatment often requires not only each of these treatments, but also a good combination of them. The dermatologist must have the knowledge and experience as a physician to provide immunotherapy as well as ointments.

### 8.1. Topical Therapy, Phototherapy, and Oral Therapy

Topical therapy includes not only the application of steroid ointments and vitamin D3 ointments, but also moisturizers and salicylic acid Vaseline [38,39]. It is important to apply a larger amount of the product externally. It is the basis of all treatment, although it is unlikely to be cured by topical therapy alone. Because of its common use, dermatologists should be familiar with its treatment.

Phototherapy may be used in combination with topical therapy when topical therapy fails to improve the condition. Irradiating lesions with ultraviolet light weakens the immune system and promotes symptom improvement. The characteristics differ depending on the wavelength of ultraviolet radiation used, including topical PUVA therapy [40,41,42] and narrowband UVB therapy. A combination of topical and phototherapy is often effective. The dose that can be increased varies from person to person and from site to site. If the dose cannot be increased to a certain level, it is necessary to consider other treatment options. Randomized controlled trials have also shown the benefit of phototherapy [43].

Oral therapy should be considered when topical therapy or phototherapy is ineffective or when arthritis is present. Biotin, etretinate, cyclosporine, methotrexate, salazosulfapyridine, or apremilast are often used [44,45,46,47,48,49]. Each oral therapy may cause a variety of side effects, including liver and kidney dysfunction, hypertension, blood cell loss, vomiting, and diarrhea. Detailed explanation to the patient and regular follow-up with blood sampling are often required. Apremilast has been tested in clinical trials or retrospective studies, and significant benefits have been obtained [50,51,52].

### 8.2. Biologic Therapy

Biologic therapy should be considered for patients who have had an inadequate response to existing therapy, who have a chronic course of moderate or severe disease, or who have complications of joint symptoms. Guselkumab (Tremfya^®^) was launched in November 2018 in Japan and improves symptoms by inhibiting IL-23, which is involved in the maintenance and proliferation of helper T (Th) 17 cells involved in neutrophil activation and pustule formation in PPP. Several cytokines are thought to be intricately involved in PPP and to cause symptoms [53]. However, oral medications and biologic agents should be used only after the focal infection is under control. Infected foci may become apparent during treatment with biologics. In such cases, the combined treatment of infected foci may lead to a cure of the PPP condition. Treatment with biologics also generally requires a follow-up, including periodic blood draws and tuberculosis. Detailed patient instructions and periodic testing will be required.

A randomized controlled trial of another biologic, anakinra (an IL-1 receptor antagonist), was conducted but did not yield significant results [54,55]. Clinical trials for spesolimab, a novel anti-interleukin-36 receptor antibody, have also been conducted. Favorable results have been obtained [56].

## 9. Latest Research

### 9.1. Single-Cell Analysis in PPP

Single-cell RNA sequencing (RNA-Seq) of lesions and PBMCs from PPP patients in the APRICOT clinical trial (Adaptive Placebo-Controlled Trial) showed elevated Th2 genes in lesions [57]. In PBMCs, memory CD4+ T cells had a Th17 phenotype. Thus, PPP T cells showed a complex activation pattern [58]. The present results have indicated systemic abnormalities in PPP [59]. Th17/Th2 cells have been reported to be IL-4/IL-17-producing in peripheral blood and from BAL (Bronchoalveolar lavage) of asthmatic patients [60,61]. Th17 cells leaning toward Th2 were suggested to be more pathogenic than Th17 cells [62,63]. Th17 cell plasticity is also associated with tobacco [64]. This suggests that Th17/Th2 cells detected in the peripheral blood and lesions of PPP patients are deeply involved in the pathogenesis of the disease. Since both Th17 and Th2 cells are closely associated with B cells, the involvement of autoantigen-reactive B cells in these pathologies is also assumed.

### 9.2. B Cells as a Source of IL-23

Thus, based on its pathogenetic hypothesis, IL-23 occupies an important position in PPP. Dendritic cells and macrophages have long been known to produce IL-23, but recently we have found that B cells also produce IL-23. We found that autoreactive B cells reacting to an autoantigen/nuclear antigen, produce a variety of cytokines [65]. We found that B cells produce not only the well-known proinflammatory cytokine IL-6 and the inhibitory cytokine IL-10, but also inflammatory cytokines such as IL-23 and inhibitory cytokines such as IL-35. Inflammatory cytokines such as IL-6 and IL-23 are produced mostly by B cells with high affinity autoantibodies. Conversely, B cells that produce inhibitory cytokines such as IL-10 and IL-35 are produced mostly by B cells with low affinity autoantibodies.

In considering B cells, cell–cell interactions with T cells are inseparable. We found that inflammatory cytokines produced by B cells induce T cells to differentiate into Th17 cells, and conversely, inhibitory cytokines produced by B cells induce Treg cells to differentiate into Treg cells. Thus, we reported the importance of B cells [66], especially cytokines produced by B cells, as factors regulating T cells. In a mouse model of autoimmune diseases, it was found that B cells producing inflammatory cytokines promote the disease, while B cells producing inhibitory cytokines suppress the disease.

Using unique microspace-based techniques and methodologies, the function and role of self-reactive B cells was clarified. The problems associated with this functional analysis were solved using a new and unique method established through medical-engineering collaboration research [67,68]. In this study, by analyzing autologous antigen-responsive B cells using micro-space formed on a microchip, it was possible to detect cytokines produced by autologous antigen-responsive B cells and to examine their ability to produce cytokines when autologous antigen-responsive B cells interact with other cells. It is expected that analysis using this approach will reveal the function of self-antigen-reactive B cells, which have been a black box. The new findings from this study are expected to be applicable to other autoimmune diseases and may create a new etiology for autoimmune diseases as a whole [69,70].

This study suggests that autoantigen-reactive B cells suppress fibrotic lesions when their affinity is low and exacerbate them when affinity is high, and that these functions are exerted via cytokine production from B cells. B cells have a variety of functions and play an important role in autoimmune diseases. In particular, self-antigen stimulation via B cell receptors induces the activation of autoreactive B cells and cytokine production, which in turn may play a major role in the formation and progression of the disease. However, direct investigations into the function of autoreactive B cells, including their reactivity to self-antigens, cytokine-producing capacity, and interactions with other cells, have not been conducted at all due to technical difficulties. The present analysis using these new methods has revealed the function of autoantigen-reactive B cells, which had been a black box. These new findings are expected to be applicable to other autoimmune diseases, including PPP, suggesting the possibility of creating a new etiology for autoimmune diseases as a whole, including neutrophils [71].

These findings may be true not only for autoimmune diseases but also for PPP. Anti-IL-23 antibody suppresses the differentiation and proliferation of Th17 cells by inhibiting IL-23 produced by B cells as well as dendritic cells and macrophages, thereby improving the skin rash on the palmoplantar surface. One of the mechanisms of action of guselkumab is that it exerts its effect by “inhibiting T cell-B cell interactions”.

## 10. Possibility to Be Effective for Other Autoimmune Diseases

From the above, there is no doubt that the anti-IL-23 antibody, guselkumab, is an effective treatment for PPP. In fact, significant results have been obtained in clinical trials. The pathogenesis hypothesis of PPP suggests that IL-23 is involved in the regulation of lymphocytes responsible for acquired immunity, such as B cells and T cells, suggesting that anti-IL-23 antibodies may be useful in other autoimmune diseases. Guselkumab, an anti-IL-23 antibody, is currently being investigated for systemic sclerosis in Japan (NCT04683029). The findings for PPP suggest that it may also be useful for systemic scleroderma, which would be a great blessing for patients.

## 11. Conclusions

PPP is a pustular disease that produces sterile pustules. However, the pathogenesis involves not only neutrophils but also B and T cells. Therapy targeting IL-23 may improve the pathogenesis of PPP not only by targeting Th17 cells, but also by affecting B cells and neutrophils. Findings for PPP suggest that targeting IL-23 may be useful not only in neutrophilic diseases but also in autoimmune diseases. The accumulation of future findings is expected.

## Figures and Tables

**Table 1:** Results of a Phase II, III Randomized Controlled Trial of guselkumab for PPP in Japan.

	Domestic Phase II Study [34]			Domestic Phase III Study [35]		
	Guselkumab Group (*n* = 25)	Placebo Group (*n* = 24)	*p*-Value	Guselkumab Group (*n* = 54)	Placebo Group (*n* = 53)	*p*-Value
Change from baseline in PPPASI	−10.2	−6.4	0.009	−15.3	−7.6	<0.001
Change from baseline in PPSI	−3.3	−1.8	0.03	−4	−2	<0.001
Number (%) of PPPASI-50 responders	15 (60)	5 (21)	0.009	31 (57.4)	18 (34.0)	0.02
